# Bitemporal Lobe Cysts

**DOI:** 10.5334/jbsr.3173

**Published:** 2023-08-09

**Authors:** Amine Naggar, Youssef Omor, Rachida Latib

**Affiliations:** 1 Ibn Sina university hospitals Center, IN

**Keywords:** radionecrosis, radiotherapy, nasopharyngeal carcinoma, central nervous system, MRI

## Abstract

**Teaching Point:** Cystic brain necrosis is a rare but severe post-radiation complication; the late post-radiation context, the temporal location, and the MRI features can suggest the diagnosis.

## Case History

A 55-year-old patient, with a history of an undifferentiated nasopharyngeal carcinoma, eight years ago, benefited from 3D conformal radiation therapy (70 Gray at 2 Gray per fraction) and concomitant Cisplatin-based chemotherapy. The patient remained under control for years before being lost to follow-up.

The patient then presented for headaches. dizziness, and behavioral changes. The physical examination showed no notable anomaly, and blood investigations were unremarkable.

**Figure 1:**
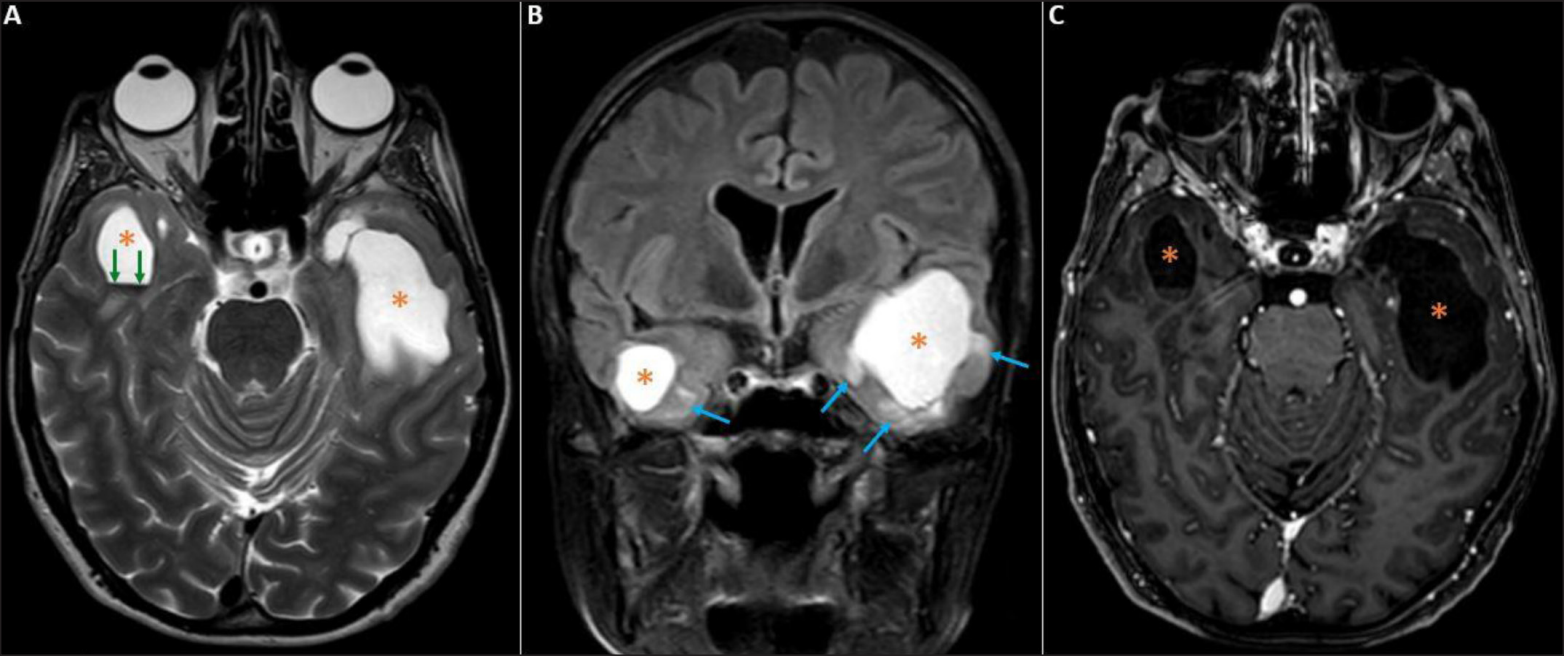


A brain magnetic resonance imaging (MRI) was performed, showing bitemporal lobe cyst-like lesions (Figure 1A–C, asterisks), with an oval shape and well-defined lobulated contours, manifesting an isointense signal on T1, hyperintense on T2 (Figure 1A), T2 FLAIR (Figure 1B), and T2 Fat-Sat, containing a T2-hypointense horizontal level of hemosiderin deposits (Figure 1A, green arrows), without diffusion restriction, and with no perceived enhancement on post-contrast (Figure 1C). The lesions were surrounded by edema (Figure 1B, blue arrows), and lead to a slight mass effect on adjacent structures but without associated cerebral herniation (Figure 1), suggesting a bitemporal lobe cystic radionecrosis.

Cystic brain radionecrosis is a rare and grave late complication of radiotherapy for cervical and intracranial neoplasms [1].

In the case of nasopharyngeal carcinoma, a cancer for which radiotherapy is the main treatment, the temporal lobes are inevitably included in the irradiation field, given their proximity to the base of the skull, which explains the high incidence of temporal localization of radionecrosis [1].

The mean latency between radiotherapy and brain cystic radionecrosis is 9.2 years. It is often asymptomatic; however, when it becomes voluminous, it may lead to mass effect and occurrence of non-specific symptoms. The most frequent symptoms are dizziness, headaches, memory impairment, and secondary seizures [1].

On MRI, cystic radionecrosis presents as cyst-like lesions, hypointense on T1, hyperintense on T2 and T2 FLAIR, without diffusion restriction, and presenting sometimes a discrete peripheral enhancement. They can be single or multiple, surrounded by perilesional edema which is hyperintense on T2 and T2 FLAIR. These lesions have the tendency to progressively increase in size, leading consecutively to mass effect [1].

The differentiation between tumoral recurrence and radionecrosis is fundamental. The late post-radiation context, the temporal location and the MRI aspect are clues to the diagnosis; nevertheless, histology remains the best diagnostic tool [1].

The therapeutic arsenal in case of symptomatic cystic necrosis includes, mainly, drainage of the cysts, and surgical resection. Conservative treatment relying on steroids can be prescribed temporarily for minimally symptomatic patients to reduce their symptoms [1].
